# Prognostic Factors in Salivary Gland Malignancies: A Multicenter Study of 229 Patients from the Polish Salivary Network Database

**DOI:** 10.3390/jcm14238527

**Published:** 2025-12-01

**Authors:** Jarosław Markowski, Wioletta Pietruszewska, Ewelina Bartkowiak, Bogusław Mikaszewski, Dominik Stodulski, Paweł Burduk, Katarzyna Radomska, Izabela Olejniczak, Aleksandra Piernicka-Dybich, Małgorzata Wierzchowska, Alicja Chańko, Daniel Majszyk, Antoni Bruzgielewicz, Patrycja Gazińska, Małgorzata Wierzbicka

**Affiliations:** 1 Department of Laryngology, Faculty of Medical Sciences in Katowice, Medical University of Silesia in Katowice, 40-055 Katowice, Poland; jmarkow1@poczta.onet.pl (J.M.); laryngologia@spskm.katowice.pl (A.P.-D.); 2 Department of Otolaryngology, Head Neck Oncology, Medical University of Lodz, 90-419 Lodz, Poland; izabela.olejniczak@umed.lodz.pl; 3 Department of Otolaryngology and Laryngological Oncology, Poznan University of Medical Sciences, 61-701 Poznan, Poland; ewelina.anna.bartkowiak@gmail.com; 4 Department of Otolaryngology, Faculty of Medicine, Medical University of Gdansk, 80-210 Gdansk, Poland; boguslaw.mikaszewski@gumed.edu.pl (B.M.); dstodulski@gumed.edu.pl (D.S.); 5 Department of Otolaryngology, Phoniatrics and Audiology, Collegium Medicum, Nicolaus Copernicus University, 87-100 Bydgoszcz, Poland; pburduk@cm.umk.pl (P.B.); wierzchowskam@op.pl (M.W.); 6 Department of Otolaryngology, Pomeranian University of Medicine, 70-204 Szczecin, Poland; kamernik@yahoo.pl (K.R.); alicja.chanko@gmail.com (A.C.); 7 Department of Otorhinolaryngology Head and Neck Surgery, Medical University of Warsaw, 02-091 Warsaw, Poland; majszykdaniel@gmail.com (D.M.); abruzgielewicz@wum.edu.pl (A.B.); 8 Biobank Research Group, PORT Polish Center for Technology Development Lukasiewicz Research Network, 54-066 Wroclaw, Poland; patrycja.gazinska@port.lukasiewicz.gov.pl; 9 Research Oncology, Division of Cancer Studies, Faculty of Life Sciences and Medicine, King’s College London, London SE1 9RT, UK; 10 Department of Otolaryngology, Regional Specialist Hospital Wroclaw, Research & Development Centre, 51-124 Wroclaw, Poland; wierzbicka.otolaryngology@gmail.com; 11 Faculty of Medicine, Wroclaw University of Science and Technology, 50-370 Wroclaw, Poland; 12 Institute of Human Genetics, Polish Academy of Sciences, 01-447 Poznan, Poland

**Keywords:** salivary glands, malignant neoplasms, parotid gland, submandibular gland, physical agents, chemical agents, mobile phones

## Abstract

**Background/Objectives:** The multitude of histological and genetic features of salivary gland malignancies (SGMs) hampers the ability of the doctors’ board to make a clear and quick decision on how aggressively treatment should be initiated. Despite treatment guidelines, it is difficult to determine the appropriate extent and invasiveness of surgery based on preliminary patient data. The aim of this study is to define the factors that have a significant impact on the oncological outcome of SGM treatment and to create an algorithm for finding the combinations of predictors with a particularly unfavorable impact on survival. **Methods**: A multicenter retrospective analysis was conducted on 2653 patients with salivary gland tumors (SGTs), including 229 with SGMs (parotid 204/229 = 89.1%; submandibular 25/229 = 10.9%), treated at seven Polish university departments from 2015 to 2022. All patients, except those with malignant lymphoma, underwent surgery followed by radiotherapy. Seventeen potential survival-impacting variables were analyzed: thirteen preoperative and four surgical specimens. The preoperative group aids in deciding surgical aggressiveness, while the postoperative group supports decisions on adjuvant treatment escalation. The main outcome measures were disease-free survival (DFS) and overall survival (OS). **Results**: SGMs constituted 8.63% of all SGTs, with 204 (89%) in the parotid and 25 (11%) in the submandibular glands. The average age was 63.38 years, with a male predominance (54%). Clinical and radiological signs of malignancy were reported in 45.4% and 54.6% of patients, respectively, with facial nerve palsy reported in 13%. Postoperative specimens revealed 23 histological types, and R0 resections were achieved in 168/229 cases (73%). Fifty-six patients (24.5%) died of cancer within five years. Significant survival factors included gender, urban residence, previous chemical and radiation exposure, clinical malignancy symptoms, pT-stage, pN-stage, clinical stage, and resection margins. **Conclusions:** The prognosis for SGM remains unsatisfactory, which would suggest more aggressive treatment; thus, carefully collected clinical data could support the decision-making process. Significantly worse survival has been demonstrated in the presence of unfavorable clinical factors, so defining new elements of medical history may be a step towards improving treatment outcomes.

## 1. Introduction

Salivary gland malignancies (SGMs) constitute 10% to 30% of all salivary gland tumors (SGTs), depending on the source of data, with the percentage increasing along with patients’ age [1,2]. SGMs are extremely diverse and heterogeneous in terms of their histopathology, which results from the complex embryogenesis of salivary glands [3,4]. As the appearance of SGMs is rare, there are few experiences describing the treatment outcomes in large groups of patients. The multitude of histological and genetic features does not make it easier for the doctors’ board to make a clear and quick decision on how aggressively treatment should be initiated. This in turn has the consequence that often only recommendations with limited evidence can be made. The most important are the National Comprehensive Cancer Network (NCCN) guidelines of 2023 [5], the American Society of Clinical Oncology (ASCO) guidelines of 2021 [6,7], the European Society for Medical Oncology (ESMO) guidelines of 2022 [8] and the British National Multidisciplinary guidelines of 2016, but all have some strengths and limitations [9].

The prognosis for SGMs depends on several factors, including the histological subtype, grade, and stage of the disease. The literature reports survival rates ranging from 19.6% to 84.7% [10]. This wide range underlines the need for a better understanding of prognostic factors; nevertheless, due to the rarity of these cancers, formulating treatment algorithms and counseling patients is often challenging. Thus, the availability of reliable predictive algorithms is helpful for evidence-based clinical decision-making [10]. Studies using data of SGC patients from the Surveillance, Epidemiology, and End Results (SEER) database have developed a predictive nomogram model for overall survival (OS) and cancer-specific survival (CSS) and have examined variables influencing survival. The SEER database, derived from cancer registries, collects high-quality, multicenter, large-sample clinical data on patient demographics, histology, stage of cancer at the time of diagnosis, the first course of treatment, and the survival time, providing a broad and reliable approach to the study of rare tumors [11]. Multiple studies using both institutional cohorts and population-based datasets have shown that age, sex, tumor site, AJCC stage, T and N classification, histologic subtype, tumor grade, treatment modality, and surgical margin status are associated with overall survival in salivary gland malignancies. These findings have been consistent across decades of research, from classical series to contemporary analyses [12,13]. A novel postoperative nomogram and risk classification system for individualized estimation of survival among patients with SGM enables individualized survival prediction and risk stratification, prompting vigilance in high-risk subgroups [14]. Other studies using data on SGM patients from the SEER database have presented predictive nomogram models for OS and CSS in SGM subtypes, such as acinar cell carcinoma, salivary duct carcinoma, adenoid cystic carcinoma, and squamous cell carcinoma [15,16,17,18,19]. In summary, an accurate predictive algorithm is paramount for assessing survival among patients with SGM, and a well-constructed nomogram plays an important role in this regard. The results suggest the superiority of nomograms over AJCC staging in predicting both 3- and 5-year survival in SGM [10,11,20].

However, the SEER database does not capture all variables which are thus not included in developing the nomograms. Data at patients’ presentation tend to be quite sparse in databases and do not reflect exposure to harmful agents in the contemporary world. Identified risk factors for SGM development comprise ultraviolet radiation, ionizing radiation, viral infections, nicotine, and alcohol consumption [1,2]. In this cohort, ‘radiation exposure’ denotes two chart-documented categories: ionizing radiation (prior therapeutic radiotherapy; secondarily, occupational exposure) and ultraviolet (UV) exposure (chronic occupational/recreational or artificial UV), recorded only when explicitly documented.

Still, little is known about the role of other potential industrial risk factors, such as widespread use of mobile phones and environmental pollution, which are difficult to link to carcinogenesis in SGM. Thus, despite the treatment guidelines, it is difficult to determine how extensive and mutilating surgery would be appropriate based on preliminary patient data. We hypothesized that carefully collected clinical data could support the decision-making process.

The primary aim of the study was to identify epidemiological and tumor-related factors associated with disease-free survival (DFS) and overall survival (OS) in salivary gland malignancies. A secondary, exploratory aim was to assess whether combinations of these factors show an additive adverse association with outcomes. Given the extent of missingness and event counts in some strata, we did not construct a multivariable prognostic algorithm in the present analysis; this is now acknowledged in the Limitations.

## 2. Materials and Methods

The study comprised 229 (8.63%) SGM out of 2653 SGT patients diagnosed and treated in the years 2015–2022 in 7 University Polish Departments. The patients’ history, along with reports on the course of surgery, HP examination, and follow-up data, were analyzed. Approval of the Bioethics Committee No 666/23 has been obtained to perform the study entitled: “Characteristics of salivary gland tumors from clinical, epidemiological and omics perspective”, Assessing committee; Bioethical Committee of the Karol Marcinkowski Medical University in Poznan, Poland. The study was performed in accordance with the Declaration of Helsinki on biomedical research involving human subjects. The assumption of the study was that each patient received adequate treatment in one of seven University Departments. All the patients were treated surgically, and then, after the Multidisciplinary Board decision, they were sent for adjuvant treatment (radiotherapy or radiochemotherapy).

We distinguished primary salivary gland SCC from metastatic SCC to intraparotid/neck nodes using predefined criteria: no history or concurrent clinical evidence of cutaneous or mucosal SCC; dermatologic and ENT examination including the scalp/face/auricle and oropharynx; contrast-enhanced imaging reviewed for dermal/subdermal primaries and intraparotid nodal disease; operative reports indicating nodal vs. parenchymal localization; pathology re-review to exclude high-grade mucoepidermoid carcinoma or salivary duct carcinoma with squamoid features and to seek an in situ ductal component. Cases not meeting criteria for primary SCC were classified as metastatic SCC. Indeterminate cases were labeled SCC-NOS (indeterminate primary) and were excluded from analyses limited to primary salivary malignancies.

### 2.1. Variables

The variables used as predictors of oncological outcomes in this study were divided into two groups: 13 variables obtained preoperatively and an additional 4 variables obtained from the surgical specimen examination. The first group would provide decision-making support regarding the aggressiveness of surgical treatment. The second group was intended to further support decisions on the escalation of adjuvant treatment.

The analysis of preoperative variables included the distribution of age and gender (Table 1). The exposure to adverse factors, i.e., chemicals, radiation, and smoking, was categorized as yes/no. Exposure was abstracted from oncology notes, radiotherapy summaries, and occupational histories; routine diagnostic imaging was not considered exposure. Owing to >40% missingness, analyses were designated as exploratory and performed complete-case analyses. ‘No data’ was not treated as an exposure level, and exposure variables were not entered into multivariable models.

A separate research question was the attempt to correlate the occurrence of SGM with cell phone use. We categorized the use of cell phones with the following daily exposure: up to 1 h/24 h, 1–2 h/24 h, 2–4 h/24 h, and >4 h per 24 h. We also analyzed the duration of cell phone use (>1–5 years, >5–10 years, >10 years) and use of headphones on the tumor side/opposite side/interchangeably. The place of residence was categorized as A—rural areas, B—towns <100 thousand inhabitants, C—towns 100÷500 thousand, D—towns >500 thousand.

Clinical manifestations of malignancy were categorized as the presence of at least one of the following symptoms: rapid tumor growth or acceleration of its growth after a long period of stable size, pain, paresis/facial nerve palsy, skin infiltration (ulceration), lesions at the duct orifice, bloody discharge from the orifice, fixation of a previously mobile tumor, or concomitant enlarged cervical lymph nodes. The presence of preoperative facial nerve palsy was analyzed separately and was categorized as absent/present. Tumor location was described according to the European Salivary Gland Society (ESGS) five-level system (levels I–V). For parotid tumors, level I corresponds to the lateral superficial lobe and level V to the deep, skull base–adjacent region; intermediate levels (II–IV) represent progressively deeper/medial segments. We coded the anatomical level recorded by the operating surgeon and/or in the imaging report.

Tumor size was categorized into three groups: <2 cm, 2–4 cm, and >4 cm. Clinical stage of the malignancy (pT-stage, pN-stage) was classified according to the 8th edition of the AJCC staging system. Radiological features of malignancy were assessed by radiologists as present/absent in preoperative computed tomography or magnetic resonance imaging [21].

Site-specific survival curves were generated as unadjusted, descriptive, complete-case displays; no site effect was modeled due to heterogeneity and the risk of confounding by indication.

The agreement between fine needle aspiration biopsy (FNAB) findings and final histology was assessed. For FNAB evaluation, the Milan System for Reporting Salivary Gland Cytopathology (MSRSGC) was used [22]. Consistency of FNAB with the final histology was categorized as yes, partial, or no.

All patients except for those with a diagnosis of lymphoma underwent surgical treatment. The type of parotidectomy and the type of neck dissection were recorded according to ESGS and AJCC 8th edition classifications, respectively, and are shown in Table 1. In the entire cohort (n = 229), 25 patients (10.9%) underwent complete excision of the submandibular gland with preservation of the facial nerve. In the remaining 204 parotid cases, we performed 96 procedures (41.9% of the whole cohort; 47.1% of parotid tumors) involving the superficial lobe, i.e., superficial parotidectomy (partial superficial parotidectomy, PSP; lateral conservative parotidectomy, LCP); 69 procedures (30.1%; 33.8%) of subtotal or total conservative parotidectomy with facial nerve preservation (subtotal conservative parotidectomy, STCP; total conservative parotidectomy, TCP); and 39 procedures (17.0%; 19.1%) of total or near-total parotidectomy with facial nerve resection (total radical parotidectomy, TRP; total semiconservative parotidectomy, TSCP). For clarity, ESGS denotes the European Salivary Gland Society, and “VII” refers to the facial nerve.

Postoperative variables included data from the histological examination of the surgical specimens. In all cases, resection margins (R0, R1, R2 resection) and tumor morphology were assessed according to the 2017 WHO Classification of Salivary Gland Tumors [23].

The overall survival (OS) and disease-free survival (DFS) were the primary outcome measures.

### 2.2. Statistical Analysis

Within the framework of statistical analysis, Kaplan–Meier survival curves have been determined for all treated patients, taking into account OS and DFS. Moreover, Kaplan–Meier curves have been determined considering the breakdown of treated patients from the perspective of selected features, such as age, gender, and the place of residence. Tumor location (parotid gland with enlisted levels or submandibular gland), tumor extensiveness in centimeters, smoking, exposure to chemical agents and radiation, time of using cell phone per 24 h, the head side/ear at which the phone was used, clinical and radiological manifestations of malignancy, facial nerve paresis, T, N grading, stage of clinical progression and variables obtained from the surgical specimen examination. For each predictor, survival analyses were based on complete-case data; participants with missing values for that predictor were excluded from that specific comparison. ‘No data’ was not treated as a category. Variables with >30% missingness were prespecified as exploratory and are reported descriptively without inferential claims. The comparison of two curves was made by means of the log-rank test; in the case of more than two curves, the chi-square test was used.

## 3. Results

In the analyzed group of 229 patients with primary SGM, women accounted for 45.4% (104 cases) and men for 55.6% (125 cases). The mean age of the treated patients was 63.38 years (range, 21–97 years). With respect to tumor location, the parotid gland predominated (204 cases), followed by the mandibular gland (25 cases). Clinical and pathological characteristics of the group are presented in Table 1, Table 2 and Table 3. For each analysis, we report the effective sample (n/N) after excluding cases with missing values.

**Figure 1:**
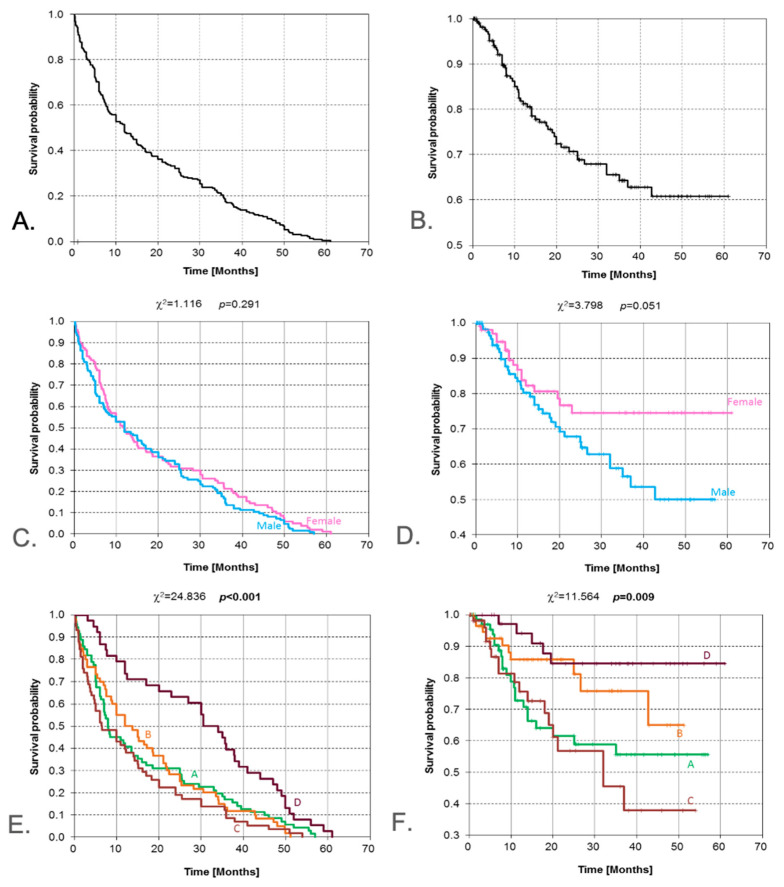
Kaplan–Meier estimate for the following: DFS (**A**) and OS (**B**) for all surgically treated patients; DFS (**C**) and OS (**D**) according to gender/sex (result of log-rank test); DFS (**E**) and OS (**F**) according to the population size of patients’ places of residence (result of chi-square test). A—rural areas, B—towns with less than 100 thousand inhabitants, C—cities with a population of 100 ÷ 500 thousand, D—cities with a population exceeding 500 thousand. DFS—disease-free survival; OS overall survival. Vertical lines indicate censoring observations.

Detailed survival analysis was performed; among those who died within 5 years of follow-up (56 patients in total, i.e., 24.5%), the longest observed survival amounted to 42.7 months. During the postoperative follow-up period, death due to malignancy within 5 years from surgery occurred in 56 patients, that is 24.5%. In this group, eight patients had survival times exceeding 2 years. Among patients with censored survival times (no signs of recurrence were disclosed at the last follow-up visit), the longest survival time of 61 months was recorded for one patient. A total of 23 patients had survival times of more than 4 years, and for 22 patients, the survival time exceeded 3 years (Figure 1A, B). Based on the results of the log-rank test, no statistically significant differences were found as concerns the pattern of Kaplan–Meier survival curves (*p* = 0.291). We added a summary of survival analyses in Table 4.

### 3.1. Gender

The log-rank test result (*p* = 0.051) is close to the conventional threshold for significance (*p* = 0.05, which, in conjunction with the visual assessment of the overall survival (OS) curves, suggests that females are in a more advantageous position. No deaths occurred in the group of surgically treated females beyond 23 months after surgery. All follow-up times longer than 23 months are censored observations (Figure 1C,D).

### 3.2. Place of Residence

The inhabitants of rural areas were most frequently affected (Table 1). The result of the chi-square test (*p* < 0.001) assessing differences in DFS curves with respect to the population size of the place of residence indicates that residents of large cities have the greatest likelihood of survival in the subsequent months. The median survival for those subgroups ranges from approximately 6 to 14 months, whereas for patients living in cities with >500,000 inhabitants, it is more than 30 months (Figure 1E,F). As indicated by the results of the chi-square test, the pattern of OS curves is significantly different (*p* = 0.009), depending on the population size of surgical patients’ places of residence. As indicated by the curves, the most advantageous position appears to be that of patients living in cities with >500,000 inhabitants.

### 3.3. Exposures

**Figure 2:**
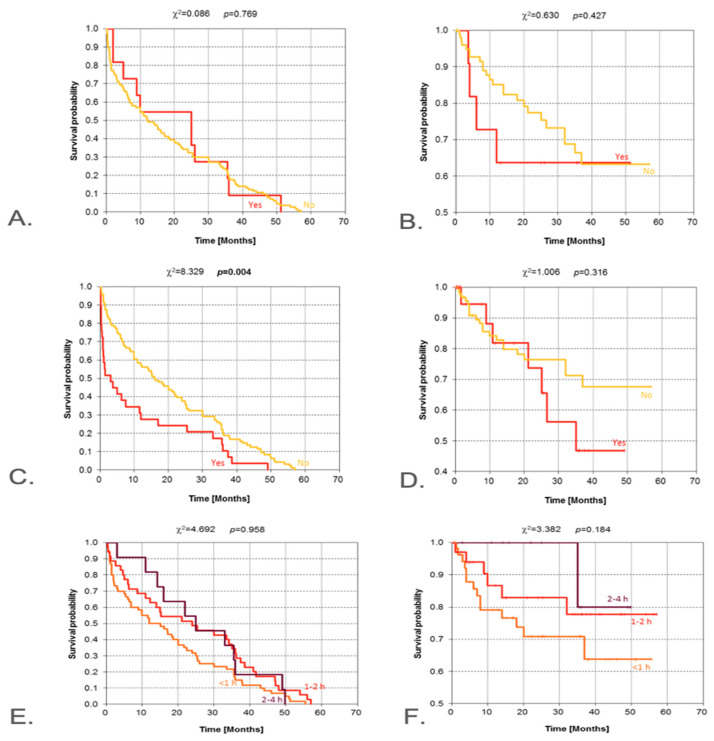
Kaplan–Meier estimate for the following: DFS (**A**) and OS (**B**) according to the exposure to chemicals; DFS (**C**) and OS (**D**) according to radiation exposure; DFS (**E**) and OS (**F**) according to the time of using cellular phones per 24 h. DFS—disease-free survival; OS—overall survival. Vertical lines indicate censoring observations; result of chi-square test. Kaplan–Meier disease-free survival by documented prior ionizing radiation (complete-case; n/N shown). Exploratory analysis: ‘no data’ excluded. UV exposure not analyzed due to missingness/confounding.

The details of the cohort with respect to smoking habits, alcohol consumption, exposure to chemicals, and radiation exposure are reported in Table 1. Survival time was analyzed according to exposure to smoking, alcohol consumption, chemicals, and radiation. The result of the log-rank test indicates a significant difference between Kaplan–Meier survival curves (*p* = 0.004). The curve for patients who were exposed to radiation lies much lower in comparison with the curve for surgically treated patients not exposed to radiation. Exposure to radiation significantly reduces the probability of disease-free survival over a specific time, which is particularly evident in the time range of 0 to 20 months (Figure 2A–D). We retained Kaplan–Meier curves only for documented prior ionizing radiation (complete-case; effective n/N shown on the plot). Given high missingness and risk of confounding, UV exposure was not analyzed with KM curves.

### 3.4. Tumor Location and Size

**Figure 3:**
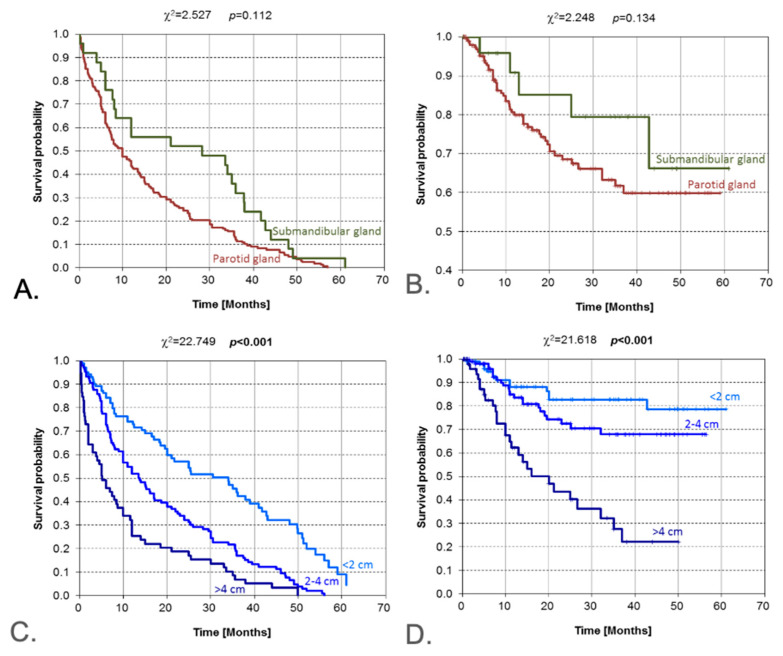
Kaplan–Meier estimate for the following: DFS (**A**) and OS (**B**) according to the salivary gland location: parotid gland versus submandibular gland; DFS (**C**) and OS (**D**) according to the tumor size. DFS—disease-free survival; OS—overall survival. Vertical lines indicate censoring observations; result of chi-square test. Overall survival by primary site (parotid vs. submandibular); unadjusted, descriptive, complete-case; n/N shown. Curves should be interpreted with caution given case-mix differences and potential inclusion of intraparotid nodal metastases within the parotid category.

Unadjusted, descriptive Kaplan–Meier curves (Figure 3; complete-case, n/N shown) suggest higher OS for submandibular versus parotid tumors; however, this pattern is plausibly explained by case-mix imbalances (histology, stage, age/competing mortality) and possible inclusion of cutaneous SCC metastases in the parotid group. We therefore refrain from inferential interpretation.

Curves showing survival with no features of recurrence according to salivary gland location and tumor diameter are shown in Figure 2A–D. The result of the log-rank test (*p* = 0.134) indicates that no significant differences exist in the pattern of Kaplan–Meier curves determined for parotid and submandibular salivary glands. In 59 patients, the size of the tumor exceeded 4 cm in diameter, and in 106 patients, it was in the range of 2–4 cm, while in an additional 57 patients, the tumor size was less than 2 cm. Curves showing survival with no features of recurrence differ significantly from the perspective of tumor size, which is manifested in high statistical significance (*p* < 0.001). The median survival (50% probability) for patients with tumor size <2 cm amounted to slightly over 30 months for tumor size from 2 to 4 cm, and some 20 months for the largest tumors, merely about 5 months. The results of the chi-square test indicate the existence of highly significant differences (*p* < 0.001) between Kaplan–Meier curves determined for patients when stratified by tumor size. Such a test result was mainly driven by the curve pattern for tumor sizes exceeding 4 cm. Patients from this subgroup had only a slightly over 20% probability of overall survival between 36 and 50 months.

### 3.5. Clinical and Radiological Signs of Malignancy

**Figure 4:**
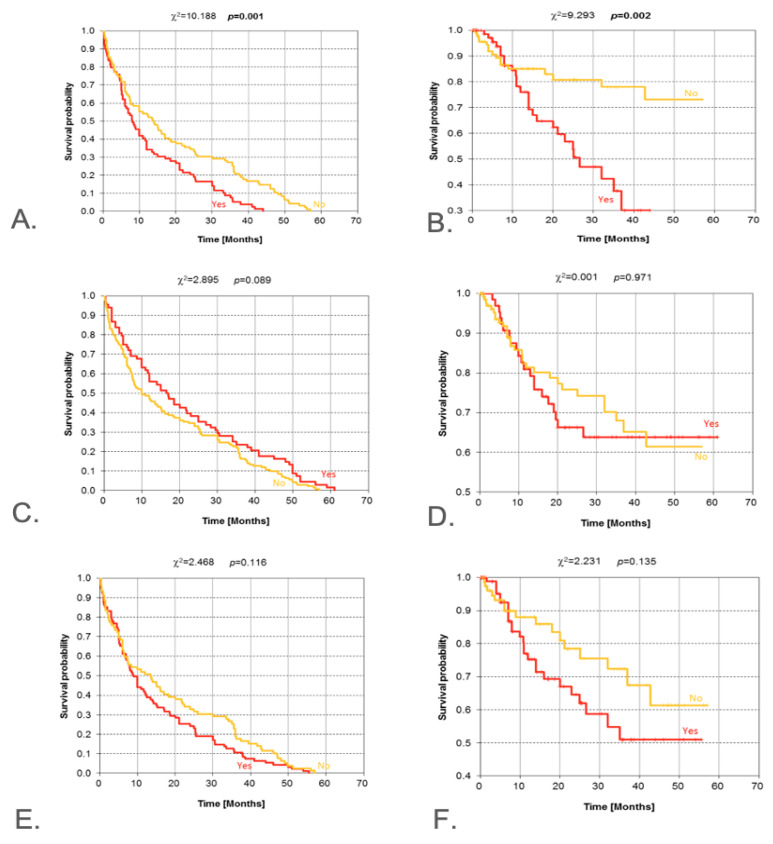
Kaplan–Meier estimate for the following: DFS (**A**) and OS (**B**) according to the presence of clinical symptoms of malignancy; DFS (**C**) and OS (**D**) according to the 7th nerve palsy; DFS (**E**) and OS (**F**) according to radiologically confirmed symptoms of malignancy. DFS—disease-free survival; OS—overall survival. Vertical lines indicate censoring observations; result of chi-square test.

Clinical signs of malignancy were reported in 79 subjects and were absent in 96 subjects. The shortest and the longest durations of symptoms were 1 and 12 months, respectively, with a mean duration of 3.87 months. Survival curves for patients with and without clinical symptoms of malignancy show different patterns, which is supported by the log-rank test (*p* = 0.001). Only for survival times up to approximately 5 months are the survival probabilities practically equal. The DFS in patients with the presence of clinical symptoms of malignancy is definitely shorter when compared to those without. The presence of symptoms of malignancy found on clinical examination is unequivocally disadvantageous from the perspective of OS. The result of the log-rank test indicates high statistical significance (*p* = 0.002) in the difference in the pattern of Kaplan–Meier curves (Figure 4A,B). Facial nerve palsy occurring before treatment was found in 22 subjects and absent in 142 subjects, but the differences in survival time of patients with and without paresis/facial nerve palsy were not significant (Figure 4C,D). Radiologically confirmed symptoms of malignancy were found in 95 subjects and were absent in 79 subjects. Survival of patients with radiologically confirmed symptoms of malignancy is presented in Figure 4E,F.

### 3.6. Tumor Advancement

**Figure 5:**
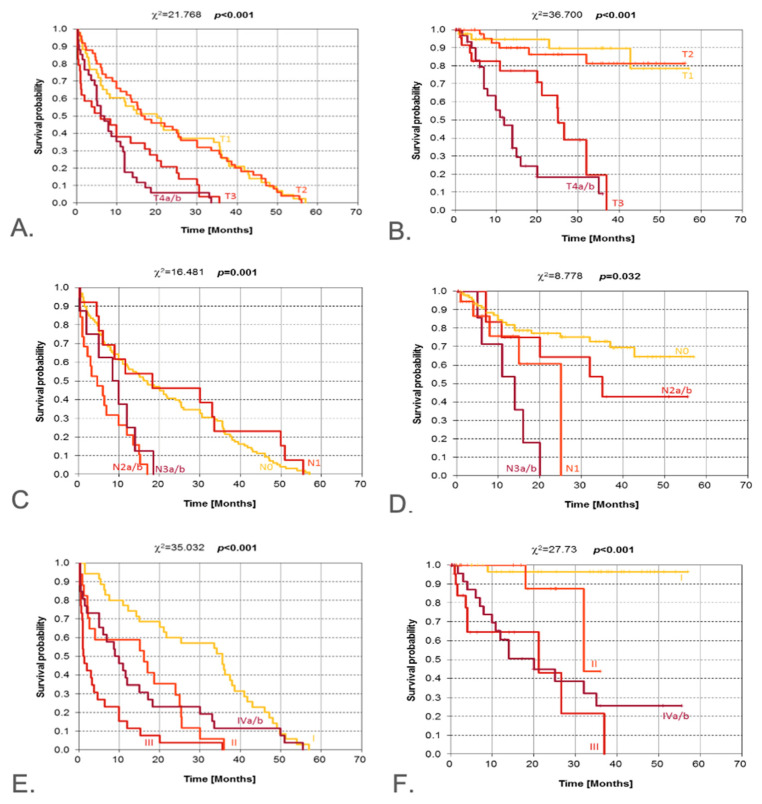
Kaplan–Meier estimate for the following: DFS (**A**) and OS (**B**) according to the pT-stage; DFS (**C**) and OS (**D**) according to the pN-stage; DFS (**E**) and OS (**F**) according to the clinical stage. DFS—disease-free survival; OS—overall survival. Vertical lines indicate censoring observations; result of chi-square test.

Curves in Figure 5A–F show disease-free survival with no evidence of recurrence according to pT, pN, and clinical stage of the disease. Curves depicting DFS in relation to pT features differ with very high statistical significance (*p* < 0.001). The curves for T1 and T2 run almost identically, which implies that patients with T1 and T2 have almost the same probability of surviving the months that follow surgery. The patterns of the two remaining curves were decisive for the test result, as these curves initially had a relatively similar pattern (up to about 10 months); after 10 months, the T4a/b curve appears to fall steeply, indicating reduced chances of disease-free survival in the surgically treated patients with this T category.

OS curves differ with high significance, as indicated by the chi-square test (*p* < 0.001). The curves for T1 and T2 have a very similar course; thus, the patterns of curves T3 and T4a/b were decisive for the result of the test. As expected, the probability of overall survival in the subsequent months is lowest in surgically treated patients with T4a/b.

The Kaplan–Meier curve patterns for DFS, stratified by N category, are clearly different, as indicated by the very high level of statistical significance (*p* = 0.001). Two subgroups of curves can be distinguished: N0 and N1, and N2a/b and N3a/b. The curves for the latter subgroup show that the probability of disease-free survival decreases very rapidly and is reduced practically to zero before the 20-month threshold after surgery is reached. 

The patterns of OS curves in relation to the N category indicate substantial heterogeneity (*p* = 0.032). The surprising shape of the curve for N1 is due to a single patient in this subgroup who survived for more than 25 months after surgery. The patterns of the other curves are in line with expectations.

The result of the chi-square test indicates very high statistical significance (*p* < 0.001) for differences in DFS curve patterns when stratified by clinical stage of disease. These curves show statistically significant differences (*p* < 0.001), and the course of the curve for stage I indicates a high probability of 5-year overall survival. The shape of the curve for stage II differs significantly from that of stage I and from those of stages III and IVa/b.

### 3.7. Histopathological Findings

**Figure 6:**
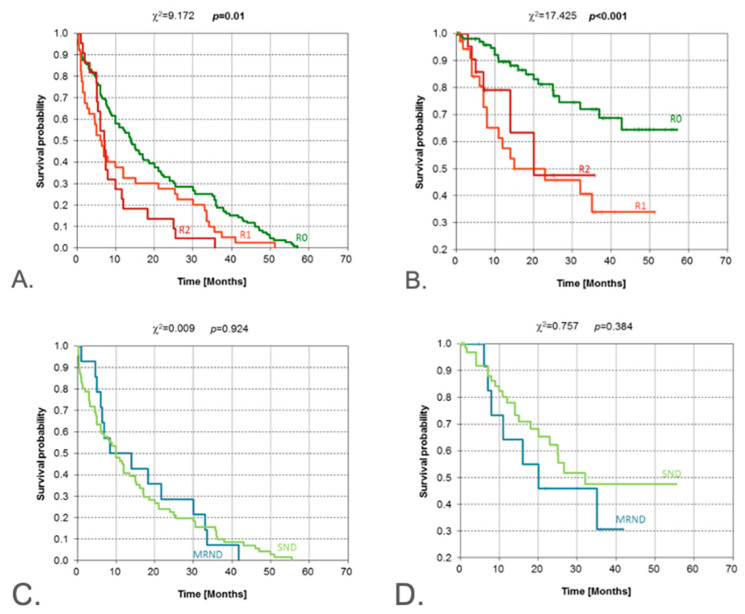
Kaplan–Meier estimate for the following: DFS (**A**) and OS (**B**) according to the resection margins; DFS (**C**) and OS (**D**) according to the type of neck dissection. DFS—disease-free survival; OS—overall survival. Vertical lines indicate censoring observations; result of chi-square test.

The most common malignant tumors in the analyzed material were as follows: squamous cell carcinoma and mucoepidermoid carcinoma—30 cases of each, with extranodal marginal zone B-cell lymphoma (MALT) as the next most frequent entity (24 cases), and adenoid cystic carcinoma and acinic cell carcinoma with 20 cases each. Details are provided in Table 2. The Kaplan–Meier curve patterns for DFS, stratified by final histology, are shown in Figure 6A,B. The patterns of OS curves in relation to histopathological subtype indicate substantial heterogeneity, although the result of the chi-square test does not reach statistical significance (Figure 6B). FNAB was attempted in 123 patients; 2 were nondiagnostic (1.6%). Among the remaining 121 diagnostic FNABs that proceeded to surgery, the exact histologic subtype matched the final histopathology in 32/121 (26%), was categorized as ‘suspicious/atypical’ in 23/121 (19%) and was discordant at the subtype level in 66/121 (54%). These figures describe diagnostic yield and subtype concordance; no inferential accuracy metrics are provided.

In the reclassified dataset, mucoepidermoid carcinoma, adenoid cystic carcinoma, and acinic cell carcinoma constitute the leading primary salivary malignancies. SCC cases were subdivided into primary salivary SCC and metastatic SCC to intraparotid or neck nodes, with the latter reported separately (Table 2). Survival analyses restricted to primary salivary malignancies exclude metastatic SCC.

In reference to the size of the surgical margin, a high level of statistical significance (*p* = 0.010) was obtained with the chi-square test when comparing DFS curves. The curve patterns are in line with expectations. In light of the chi-square test results, the OS curves show highly significant differences (*p* < 0.001). Patients with R0 resection had a much higher probability of survival, even between 43 and 57 months after surgery. The curves for R1 and R2 show similar patterns.

## 4. Discussion

This analysis of SGM was performed as part of the Polish Salivary Network Database program [24], which comprises 5-year data (2015–2022) and is an extension of the Polish National Major Salivary Gland Benign Tumors Registry (SGR), kept since 2014 [25]. Despite the existing guidelines and nomograms, it is always difficult to determine how extensive treatment would be appropriate based on preliminary patient data. Each additional variable, not yet present in large databases, may be important. Therefore, in the preliminary assumptions, we included several new parameters: clinical symptoms of cancer, exposure to chemical and physical factors, and the use of mobile phones, and we sought to determine whether such detailed preliminary clinical data have an impact on the outcome.

While SEER lacks several variables of interest (e.g., environmental exposures), our retrospective multi-centre data show substantial missingness for some of these fields, limiting inference. We therefore present these as exploratory, hypothesis-generating observations and do not incorporate them into multivariable models.

### 4.1. Clinical Symptoms

A much more frequent occurrence of benign tumors in the parotid gland may lead to underestimation of the first symptoms of SGM and delay the initiation of treatment. On the other hand, the occurrence of symptoms indicating the malignant nature of the tumor is usually associated with its highly advanced stage and poor prognosis regarding survival [26]. The clinical picture of advanced SGM is typical, although some cases initially develop insidiously without such clear signs of malignancy. Facial pain and asymmetry are striking and alarming symptoms prompting the patient to seek immediate medical advice. Enlargement of lymph nodes of the neck and infiltration of the skin add to the picture of a malignant process within the salivary gland [26]. Our research has proven that a patient who has clear clinical symptoms of SGM has a much worse prognosis in terms of DFS. Interestingly, it was important to include a set of symptoms in the analysis, not just isolated facial paralysis.

### 4.2. Data from Medical Interview

A path for SGM diagnostics and treatment is well established by the panel of experts, based on a systematic analysis of scientific papers published in the years 2000–2020 [26]. The recommendations for preoperative evaluation of patients, preferred method of surgery, and indications for radiotherapy have been developed [26]. In the preoperative assessment, using imaging diagnostics (USG, contrast-enhanced CT, and/or MR) of the neck area and the primary site is recommended. The information collected in this way is of unquestionable importance, based on EBM, and is valid when making decisions. However, in an entity as difficult as SGM, additional patient medical data can routinely be included, the combination of which would influence prognosis and tendency to more/less aggressive treatment.

Our analysis of the data yielded several interesting insights. First, place of residence emerged as a variable with a significant impact on DFS and OS and has been practically unstudied in previous publications. The largest group of patients with SGM were inhabitants of rural areas. Second, better outcomes for residents of large cities may be associated with better access to medical care and specialist services, as well as higher health awareness. On the other hand, the degree of air pollution and exposure to chemicals does not appear to differ substantially between cities, towns, and villages. Exposure to environmental factors that predispose to the development of cancer is not routinely captured during standard medical history taking. We found that exposure to chemicals and radiation did not affect DFS but had an adverse effect on OS. DFS and OS according to the daily duration of cellular phone use did not differ significantly in our cohort. Findings regarding radiation exposure should be interpreted cautiously; 45.41% missing data precluded robust adjustment and increased the risk of bias. We therefore report the exposure analyses as exploratory, with complete-case denominators.

Our data are concordant with the established literature on core prognostic factors (age, stage, T/N, histology, grade, and margins). In addition, the observed survival advantage for patients living in larger cities mirrors findings by Filho et al., who reported lower mortality and longer survival in metropolitan residents compared with rural patients, potentially reflecting differences in access to specialist care and time to diagnosis [27].

### 4.3. Histology Findings

To distinguish malignant from benign lesions, FNAB has been established [28]. We found low compatibility of the result of FNAB with the final histology. It may be puzzling that the preoperative result does not match the HP in as many as 54% of the cases. Our data show a low non-diagnostic rate but limited subtype concordance, consistent with prior evidence that FNAB mainly supports benign–malignant triage rather than precise subtyping. We removed the unsupported lower-bound concordance figure and present transparent counts/denominators without additional accuracy estimates in this retrospective, multi-center cohort [29].

In the available literature, the concordance of FNAB with final histopathology HP ranges from 38% to 91.67% [30]. This variability may be explained by the many cytomorphological overlaps described between benign and malignant salivary gland entities [31]. Moreover, metaplasia, as well as cystic and degenerative changes, are common findings that add to diagnostic dilemmas [32].

For primary SGM, radical resection is the standard treatment, apart from non-Hodgkin lymphomas [32,33]. The type of treatment was not the subject of this analysis; we assumed that each patient received standardized and adequate treatment in one of seven university departments. However, the final histopathological results were important information for this study. As is well known from the literature, SGCs are rare tumors accounting for less than 1% of all cancers with 29 histologically diverse subtypes [33] and are among the most heterogeneous tumors. In our series of 229 tumors, we confirmed 23 different histological types of malignancy. The most common histologies included squamous cell carcinoma and mucoepidermoid carcinoma—with 30 cases of each (13%). The most common malignant tumor in the literature was adenoid cystic carcinoma (ACC) [1,2,34], mucoepidermoid carcinoma (MEC) [1,2,3,34,35,36] with acinic cell carcinoma (ACC) in third place [3]. Squamous cell carcinoma (SCC) was the most common SGM in New Zealand [37] and German populations [38]. Consistent with the WHO (2017), primary salivary gland SCC is rare and should only be diagnosed after thorough exclusion of a prior SCC and other salivary primaries with squamoid differentiation; many parotid SCCs reflect metastatic cutaneous SCC to intraparotid lymph nodes. Our reclassification separates primary SCC from metastatic SCC and prevents inflation of primary SCC incidence in the series. In the literature on the subject, in relation to various histologies, a whole range of factors are mentioned that predispose to obtaining a better survival result. Adjusted survival analyses stratified by treatment and staging performed with the primary outcome of OS and further stratified based on histologic subtype demonstrated unique treatment patterns and survival outcomes based on major histologic subtype [39].

Prognosis and outcomes of SGM in multivariate analyses presented in the literature constitute a mosaic of variables and results. An independent impact on OS was detectable for patient age, T, and N in the vast majority of authors. Advanced age, submandibular gland lesions, T3–4 stage, and lymph node involvement were independent prognostic factors [40], although in patients with tumors > 4 cm, adjuvant radiotherapy improved OS [14]. For specific histology, i.e., ACC, smoking, age, and gender did not correlate with OS and should not be used for prognostication [41]. The strongest favorable prognostic factors were early disease stage (stage I and II) and major salivary gland subsite with the best prognosis in the parotid gland, irrespective of disease stage, perineural invasion, or radical surgery [42]. Although patients with positive margins had poorer outcomes, after controlling for overall stage, histologic risk group, and adjuvant radiotherapy, margin status was not a factor associated with poorer DFS. In patients with close margins, low-risk and intermediate-risk histologic types, and overall pathologic stage I/II, patients who did not have adjuvant radiotherapy had comparable local control with those who received adjuvant radiotherapy [42,43]. In our study, resection margin status (R0 versus R1/R2) differed significantly with respect to both OS and DFS.

To summarize, we have determined the epidemiological factors and tumor characteristics that have a significant impact on DFS and OS, and which can be obtained before starting the treatment. In addition to well-known and scientifically proven variables, we added a combination of subsequent significant variables such as tumor size, clear clinical features of malignancy at presentation, availability of a specialist (large/small city), and currently relevant harmful factors such as exposure to physical and chemical agents but not the use of mobile phones. The weak points of the work are some data gaps in the various analyzed variables. These deficiencies did not allow for logistic regression methods and the determination of a combination of parameters with a total impact on the result. The remedy is to continue collecting material and develop a bigger, more complete group.

### 4.4. Limitations

We were unable to build a stable multivariable model (and thus a prognostic algorithm) due to gaps in several variables and limited events within some histologic and stage strata; future work will address this through standardized prospective data collection and imputation strategies where appropriate.

The pattern and extent of missing data across selected variables precluded stable multivariable modeling or nomogram development in the present analysis. Future work will use prospective standardized case report forms and consider multiple imputations when assumptions are met.

Given the retrospective design across seven centres, misclassification between primary salivary SCC and metastatic SCC is possible. We mitigated this by applying explicit clinical–radiologic–pathologic criteria (Methods) and by reporting metastatic SCC separately.

Site comparisons are sensitive to histology, stage, age, and handling of metastatic cutaneous SCC to intraparotid nodes; residual confounding and small numbers limit inference.

## 5. Conclusions

The prognosis for SGM remains unsatisfactory, which would suggest more aggressive treatment; thus, carefully collected clinical data could support the decision-making process. Significantly worse survival has been demonstrated in the presence of unfavorable clinical factors, so defining new elements of medical history may be a step towards improving treatment outcomes.

## Figures and Tables

**Table 1:** Clinicopathological features of 229 patients suffering from malignant neoplasms of salivary glands.

Clinicopathological Features	N (%)
Age (years)	21–97 (mean 63.38)
Gender	
Men	125 (55.6%)
Women	104 (45.4%)
Clinical symptoms of malignancy	79—yes
	96—no
	No data for 54 subjects
Duration of symptoms (months)	1–12 (mean duration 3.87 months)
Radiological signs of malignancy	95—yes
	79—no
	No data for 55 subjects
Preoperative facial nerve palsy	Yes—22
	No—141
	No data for 65 subjects
Postoperative facial nerve palsy	69—yes
	24—yes, partial
	111—no
	No data for 25 subjects
Duration of paresis in nerve VII	76—no nerve paresis
	50—permanent paresis
	Transient paresis:
	>7 days–1 month—8 subjects
	1–3 months—6 subjects
	3–6 months—6 subjects
	6–12 months—5 subjects
	No data for 78 subjects
Tumor size	No data for 7 subjects
<2 cm	57
2–4 cm	106
>4 cm	59
Place of residence	No data for 2 subjects
Rural area	71 (31%)
Town <100,000 inhabitants	60 (26%)
Town/city, population 100,000–500,000 inhabitants	58 (25%)
City, population >500,000 inhabitants	38 (16%)
Cigarette smoking	49-yes
	79-no
	No data for 101 subjects
Alcohol consumption	5-yes
	121-no
	No data for 103 subjects
Exposure to chemicals	11-yes
	114-no
	No data for 104 subjects
Exposure to radiation	29-yes,
	96-no
	No data for 104 subjects
Neck dissection	
SND	121
RND	5
MRND	24
No nodal surgery performed	79
Resection	
R0	168
R1	32
R2	29
Complementary treatment:	
radiotherapy	139
Chemotherapy	6
No adjuvant therapy	84
pT-stage	
T1	74
T2	75
T3	39
T4a	37
T4b	4
pN-stage	
N0	149
N1	47
N2a	5
N2b	18
N3a	3
N3b	7
pM-stage	
M0	229
M1	0
Clinical stage	
I	74
II	75
III	37
IVa	23
IVb	12

**Table 2:** The histopathological diagnosis of surgically treated malignancies (N = 229).

No.	Histopathological Diagnosis	No. of Cases	%
1	Mucoepidermoid carcinoma	30	13
2	Squamous cell carcinoma	30 including 24 (80%) primary salivary SCC and 6 metastatic SCC (20%) to intraparotid nodes	13
3	Adenoid cystic carcinoma	20	8
4	Acinic cell carcinoma	20	8
5	Carcinoma ex pleomorphic adenoma	17	7.5
6	Salivary duct carcinoma	15	6.5
7	Epithelial-myoepithelial carcinoma	11	4.8
8	Myoepithelial carcinoma	8	3.4
9	Adenocarcinoma, NOS	7	3.0
10	Small cell neuroendocrine carcinoma	6	2.6
11	Polymorphous adenocarcinoma	4	1.7
12	Basal cell adenocarcinoma	6	2
13	Intraductal carcinoma	1	0.004
14	Adenocarcinoma cribriform	2	0.008
15	Secretory carcinoma	2	0.008
16	Carcinosarcoma	3	1.3
17	Undifferentiated carcinoma	4	1.7
18	Large cell neuroendocrine carcinoma	0	0
19	Metastasis of neuroendocrine tumor	1	0.004
20	Lymphoepithelial carcinoma	2	0.008
21	Oncocytic carcinoma	3	1.3
22	Extranodal marginal zone B-cell lymphoma (MALT)	24	10
23	Metastasis of melanoma	1	0.004
24	other lymphomas	12	5.2
	Total	229	100

**Table 3:** The location of the tumor within the parotid salivary gland according to the ESGS classification (N = 229).

Location (ESGS)	Number of Patients (n)	% of Patients
I	11	4.8
II	40	17.5
I_II	45	19.7
III	9	3.9
II_III	23	10.0
I_II_III	6	2.6
I_II_III_IV	21	9.2
IV	1	0.4
I_IV	6	2.6
V	5	2.2
II_III_IV	4	1.7
III_IV_VI	10	4.4
I_II_V	5	2.2
VI	3	1.3
I_II_IV	1	0.4
I_V	2	0.9
I_II_III_IV_V	4	1.7
I_II_III_IV_VI	2	0.9
III_VI	2	0.9
III_IV	2	0.9
I_II_III_IV_V_VI	1	0.4
II_III_VI	1	0.4
Submandibular	25	10.9
Total	229	100.0

**Table 4:** Summary of survival analyses (DFS and OS).

Predictor	Levels (Summary)	Statistical Test	DFS *p*-Value	OS *p*-Value	Notes
Place of residence	Rural; small towns; cities 100–500 k; cities >500 k	Log-rank/χ^2^	<0.001	0.009	Better outcomes in large cities
Exposure: prior radiation	Yes vs. No	Log-rank	0.004	-	Significant for DFS; OS not reported as significant
Tumor size (diameter)	Ordinal increase	Log-rank/χ^2^	<0.001	<0.001	Monotonic worsening with larger size
Clinical signs of malignancy	Present vs. Absent	Log-rank	0.001	0.002	Early months especially impacted
Pathological T (pT)	T1–T4	χ^2^	<0.001	<0.001	Clear separation of curves
Pathological N (pN)	N0–N3	χ^2^	0.001	0.032	Nodal involvement worsens outcomes
Clinical stage (AJCC)	I–IV	χ^2^	<0.001	<0.001	Progressive decline by stage
Resection margins	R0 vs. R1/R2 (positive)	χ^2^	0.010	<0.001	R0 strongly favorable for OS
Gender	Female vs. Male	Log-rank	0.051	-	Borderline/not significant

Abbreviations: DFS: disease-free survival; OS: overall survival; χ^2^: chi-square test. Notes: *p*-values are from log-rank or chi-square tests, as appropriate to the Kaplan–Meier comparisons reported. Analyses used a complete-case approach; sample size varies by predictor due to missingness. “-” indicates not significant or not assessed for that endpoint.

## Data Availability

The data presented in this study are available upon request from the corresponding author. The data are not publicly available due to privacy and data protection restrictions.
